# Soft Mango Firmness Assessment Based on Rayleigh Waves Generated by a Laser-Induced Plasma Shock Wave Technique

**DOI:** 10.3390/foods10020323

**Published:** 2021-02-03

**Authors:** Nayuta Arai, Masafumi Miyake, Kengo Yamamoto, Itsuro Kajiwara, Naoki Hosoya

**Affiliations:** 1 Division of Mechanical Engineering, Shibaura Institute of Technology, 3-7-5 Toyosu, Koto-ku, Tokyo 135-8548, Japan; md19004@shibaura-it.ac.jp (N.A.); md19074@shibaura-it.ac.jp (M.M.); md16095@shibaura-it.ac.jp (K.Y.); 2 Division of Human Mechanical Systems and Design, Hokkaido University, N13, W8, Kita-ku, Sapporo-shi, Hokkaido 060-8628, Japan; ikajiwara@eng.hokudai.ac.jp; 3 Department of Engineering Science and Mechanics, Shibaura Institute of Technology, 3-7-5 Toyosu, Koto-ku, Tokyo 135-8548, Japan

**Keywords:** firmness, laser-induced plasma, mango, non-contact non-destructive excitation, Rayleigh wave propagation velocity, shock wave

## Abstract

Many methods based on acoustic vibration characteristics have been studied to indirectly assess fruit ripeness via fruit firmness. Among these, the frequency of the _0_S_2_ vibration mode measured on the equator has been examined, but soft-flesh fruit do not show the _0_S_2_ vibration mode. In this study, a Rayleigh wave is generated on a soft mango fruit using the impulse excitation force generated by a laser-induced plasma shock wave technique. Then, the flesh firmness of mangoes is assessed in a non-contact and non-destructive manner by observing the Rayleigh wave propagation velocity because it is correlated with the firmness (shear elasticity), density, and Poisson’s ratio of an object. If the changes in the density and Poisson’s ratio are small enough to be ignored during storage, then the Rayleigh wave propagation velocity is strongly correlated to fruit firmness. Here, we measure the Rayleigh wave propagation velocity and investigate the effect of storage time. Specifically, we investigate the changes in firmness caused by ripening. The Rayleigh wave propagation velocity on the equator of Kent mangoes tended to decrease by over 4% in 96 h. The Rayleigh wave measured on two different lines propagated independent distance and showed a different change rate of propagation velocity during 96-h storage. Furthermore, we consider the reliability of our method by investigating the interaction of a mango seed on the Rayleigh wave propagation velocity.

## 1. Introduction

People who work in agriculture assess fruit ripeness based on a variety of indexes such as size, color, shape, and aroma prior to harvest. Moreover, the soluble solid content, acidity, and firmness, which are related to fruit ripeness, are often used as ripeness indexes [1,2,3,4,5,6,7,8,9]. These indexes are classified using optical, biochemical, or mechanical methods.

Many approaches based on an optical method have been proposed to assess the soluble solid content or acidity of the fruit by irradiating with visible or near-infrared light [2,3,6,7,9,10,11,12,13,14,15]. Although these methods have practical applications, they are difficult to apply to fruit whose peel does not change color or does not transmit light during the ripening stage.

Biochemical methods detect aromatic volatiles and assess ripeness by gas chromatography or an electric nose [16,17,18,19,20,21]. This approach is not suitable to assess the ripeness of fruit that emits a weak odor. In addition, neither optical nor biochemical methods can assess the ripeness of fruit such as avocado, which show low soluble solid content or acidity. Consequently, many mechanical methods to assess the firmness of fruit as a ripeness index have been proposed [22,23,24,25,26,27,28,29,30,31].

In mechanical methods, dynamic characteristics such as the natural frequencies or vibration modes are assessed by contact devices such as an impact hammer, pendulum, or vibrator because the natural frequency, which is called the _0_S_2_ mode, is strongly correlated to fruit firmness [32]. However, traditional contact devices are not suitable to excite soft fruit because the excitation damages the fruit flesh. Furthermore, it is assumed that the strong viscoelastic feature of soft fruit limits excitation of the _0_S_2_ mode frequency.

Previously, we demonstrated a non-contact and non-destructive firmness assessment system by applying a shock wave generated by laser-induced plasma (LIP) as an impulse excitation force [33]. The changes to the firmness of apples during storage were assessed by monitoring the shift in the _0_S_2_ mode frequency. As the firmness decreases, the _0_S_2_ mode frequency shifts to a lower frequency band. In our LIP shock wave excitation technique, a plasma is formed by condensing a laser beam from a high-output neodymium: yttrium aluminum garnet (Nd:YAG) pulsed laser to a point near the excitation point. A shock wave is generated when the plasma expands at an ultrasonic speed. This shock wave is applied to a test piece as an impulse excitation force. The LIP shock wave excitation technique has been widely developed for assessments or non-destructive inspections of the dynamic characteristics of mechanical structures as well as for the generation of a point sound source [34,35,36,37].

Laser ablation (LA) can generate a larger excitation force than a LIP shock wave. In LA, a plasma plume with a high temperature and high density is formed by directly irradiating a high-output pulsed laser to the excitation point of a target object (solid surface). When mass *Δm* is emitted from the object at velocity *v*, the momentum, which is given by *Δmv*, acts as the impulse to the object. Many studies have applied LA to vibration or acoustic tests for mechanical structure assessments [38,39], bolt loosening detection [40], infrastructure inspection [41], damage detection [42], and mechanical property measurement in hydrogels [43]. Because LA causes sub-millimeter-sized damage on the object’s surface, it is difficult to assess its firmness via LA and maintain commercial value.

One firmness assessment for apples uses thermoelastic wave propagation on the apple surface by a laser ultrasonic (LU) technique [44]. LU uses a lower laser fluence (laser pulse energy) than LA. LU and LA both irradiate a laser beam to a target structure directly. LU is often used to inspect mechanical structures or composite materials [45,46] because it can produce a non-contact and non-destructive excitation force. However, LU has three main drawbacks. It produces a measured elastic wave with a small amplitude. It has a low signal-to-noise ratio, and it requires numerous measurements. Additionally, it can also generate LA, depending on the material of the target object, wavelength of the pulsed laser, and laser fluence. The threshold to generate LU or LA is difficult to determine, which limits the practicality of this method to assess fruit firmness.

In this paper, a Rayleigh wave is generated on the surface of a soft fruit peel using an impulse excitation force generated by a LIP shock wave technique. The flesh firmness of soft fruit is assessed in a non-contact and non-destructive manner by observing the Rayleigh wave propagation velocity. A Rayleigh wave is a type of surface elastic wave that propagates on a target object and is commonly used for the non-destructive assessment of Young’s modulus [47] or defect inspection [48,49,50]. In addition, Rayleigh wave propagation on soft gels and flesh fruit has been investigated [51,52]. The Rayleigh wave velocity is correlated to the shear elasticity (firmness) of an object, density, and Poisson’s ratio. If the changes in the density and Poisson’s ratio of the fruit can be ignored during the storage period, only shear elasticity (firmness) changes. According to a previous study [52], shear elasticity is correlated to fruit flesh firmness. We can infer that the Rayleigh wave propagation velocity decreases as shear elasticity decreases. The LIP shock wave excitation technique can improve the signal-to-noise ratio because it produces a larger excitation force compared to LU. An assessment of the firmness of soft fruit by generating a Rayleigh wave via a LIP shock wave excitation technique has yet to be reported.

Herein, we report such an assessment using mangoes as a soft fruit. First, we demonstrate that a Rayleigh wave is generated on a mango fruit by applying a LIP shock wave as an impulse excitation force. We obtain the vibration responses on each measurement point by a laser Doppler vibrometer (LDV). Then, the propagation velocity is calculated from those time responses. Second, the change in the Rayleigh wave propagation velocity is regarded as the change in firmness during storage. We assess the firmness of a mango fruit by monitoring the decreased Rayleigh wave velocity. Finally, we examine the location of the mango fruit where the Rayleigh wave should be generated to assess firmness to avoid possible bias from the large mango seed.

## 2. Materials and Methods

### 2.1. LIP Shock Wave Excitation Technique

LIP is a phenomenon where plasma with a high temperature and high density is formed by condensing a high-output pulsed laser in air with a laser fluence exceeding 10^15^ (W/m^2^). As the plasma expands to the surroundings at ultrasonic speed, a shock wave is generated. This shock wave acts on the fruit as an impulse excitation force [53].

**Figure 1:**
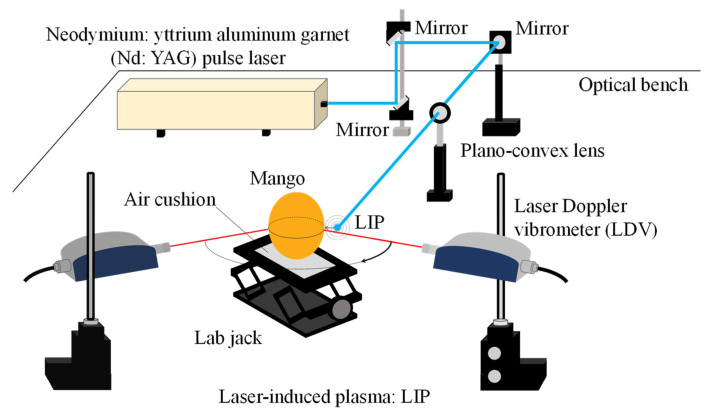
Firmness assessment system for mangoes using a LIP shock wave as a non-contact and non-destructive excitation method.

Figure 1 shows the firmness assessment system for mangoes based on the LIP shock wave excitation technique. A laser beam from a Nd:YAG pulsed laser (Surelite III-10, Continuum Inc., Milpitas, CA, USA, wavelength 1064 nm, laser beam radius: 4.75 mm, pulse width: 5 ns, maximum output: 850 mJ, and radial divergence angle: 0.25 mrad) installed on an optical bench was condensed with a plano-convex lens (SLB-20–100P, SIGMAKOKI Co., LTD., Sumida-Ku, Tokyo, Japan, focal length: 100 mm) to generate a LIP shock wave near the excitation point on mangoes. The pressure *P*^sh^(*d*) (Pa) generated by a LIP shock wave is given by [54]:

(1) $$P^{\mathrm{sh}}\left(d\right)={\left(\frac{2}{5}\right)}^{2}\frac{2\zeta_{0}^{5}}{\gamma +1}Ed^{-3}$$

where *d* (m) is a distance from the LIP generation point, *ζ*_0_ is a constant (*ζ*_0_ = 0.93), *γ* is the specific heat ratio (*γ* = 1.41), and *E* (J) is the laser pulsed energy. In this experiment, the pressure *P*^sh^(*d*) generated by the LIP shock wave is estimated as 2.9 MPa because we set the distance *d* (hereinafter referred to as the standoff distance) between the excitation point on the mango peel and the LIP generation point to 3 mm.

The vibration responses on a mango are measured by LDV (NLV-2500-5, Polytec GmbH, Waldbronn, Baden-Württemberg, Germany) in a non-contact manner and recorded by a spectrum analyzer (A/D: NI PXI-4462, National Instruments Co., Austin, TX, USA, Software: CAT-System, CATEC Inc. Taito-Ku, Tokyo, Japan). In the experiment, we placed reflection seals (approximately 3 mm × 3 mm) at each measurement point. We set the sampling frequency, the number of sampling points, and the number of trials to 204.8 kHz, 32,768 points, and 5, respectively. The mango was seated on an air cushion to ensure free support.

### 2.2. Storage Time and Measurement Paths of Mangoes

**Figure 2:**
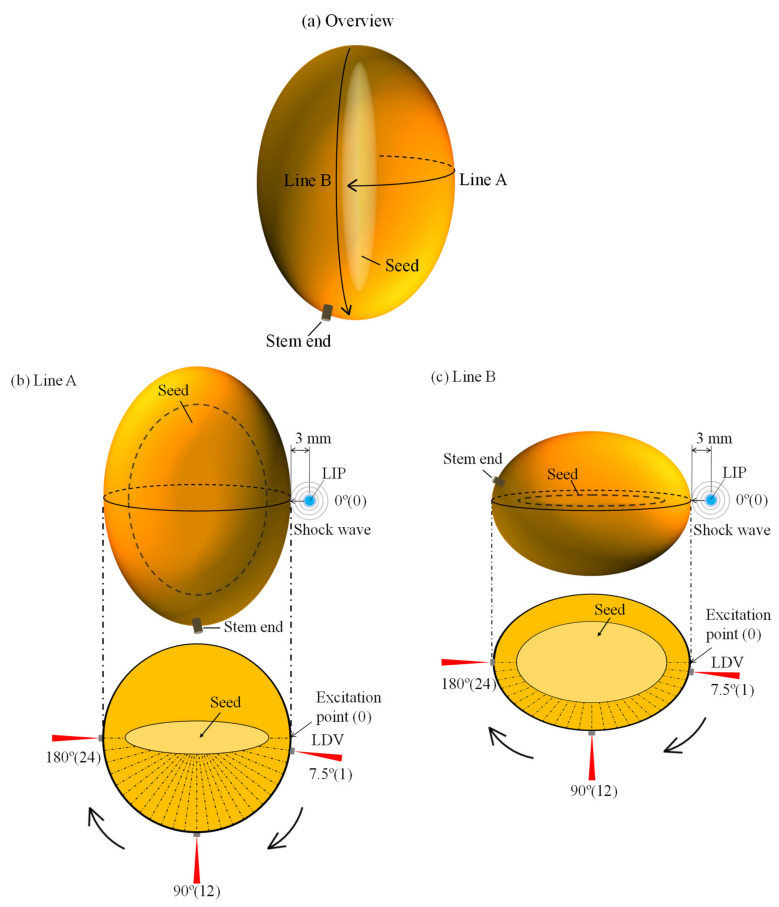
Measurement paths of the Rayleigh wave on a mango. (**a**) Overview of a mango. (**b**) Line A represents the measurement points on the equator. (**c**) Line B represents the measurement points orthogonal to the equator.

The experiment used 20 Kent mangoes as test material (Table 1). The average mass, density, and circumference (Line A, see Figure 2) were 0.457 kg, 1094 kg/m^3^, and 27 cm, respectively. Table 2 shows the storage time and measurement paths of the mangoes.

First, we considered the storage time and storage method. Mangoes 1–5 and 9–20 were used to observe the changes in the Rayleigh wave propagation velocity excited on the mango surface at 0, 48, and 96 h of storage. Mango 6 was used to observe the changes in the Rayleigh wave propagation velocity over 216 h of storage measured in 24 h increments. Here, “0 h” represents the starting time of the experiment for each mango. During the experiments, mangoes were stored at a constant temperature of 19 °C.

Next, we considered the measurement points on mangoes. Figure 2 depicts the measurement points on a mango fruit. Line A was on the equator of the mango, while Line B was orthogonal to the equator. We measured a Rayleigh wave on the equator because a fruit firmness was assessed by vibration tests to obtain the _0_S_2_ frequency [33]. We also determined Line B, which is orthogonal to the equator. In a previous study where the flesh firmness of watermelon was assessed by measuring the Rayleigh wave propagation velocity [52], the flesh thickness was shown to be correlated to the Rayleigh wave propagation velocity. The fruit flesh thickness is small on Line B because mangoes contain large seeds.

Mangoes 1–20 were assessed on Line A, and mangoes 9–20 were assessed on Line B also. Line A consisted of 24 points set in the equatorial direction on the semi-equator in 7.5° intervals (Figure 2). Line B consisted of 24 points in the orthogonal direction to the equator in 7.5° intervals, where point 0 is in the opposite direction of the stem end. For both Lines A and B, we set point 0 as the excitation point and points 1–24 as the measurement points. Laser beam from LDV was installed in the normal direction to the tangent plane containing each measurement point.

## 3. Results and Discussion

### 3.1. Measured Time History Responses and Their Power Spectra

**Figure 3:**
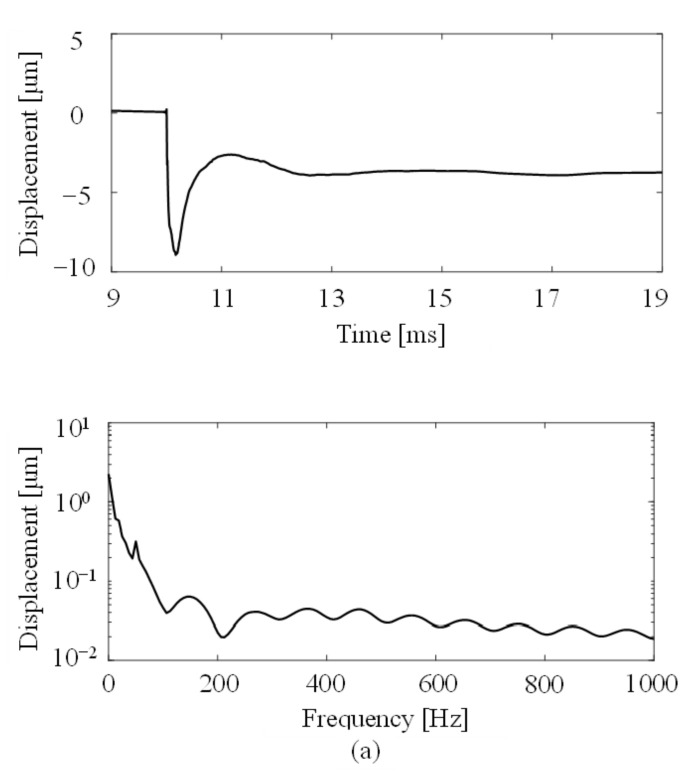

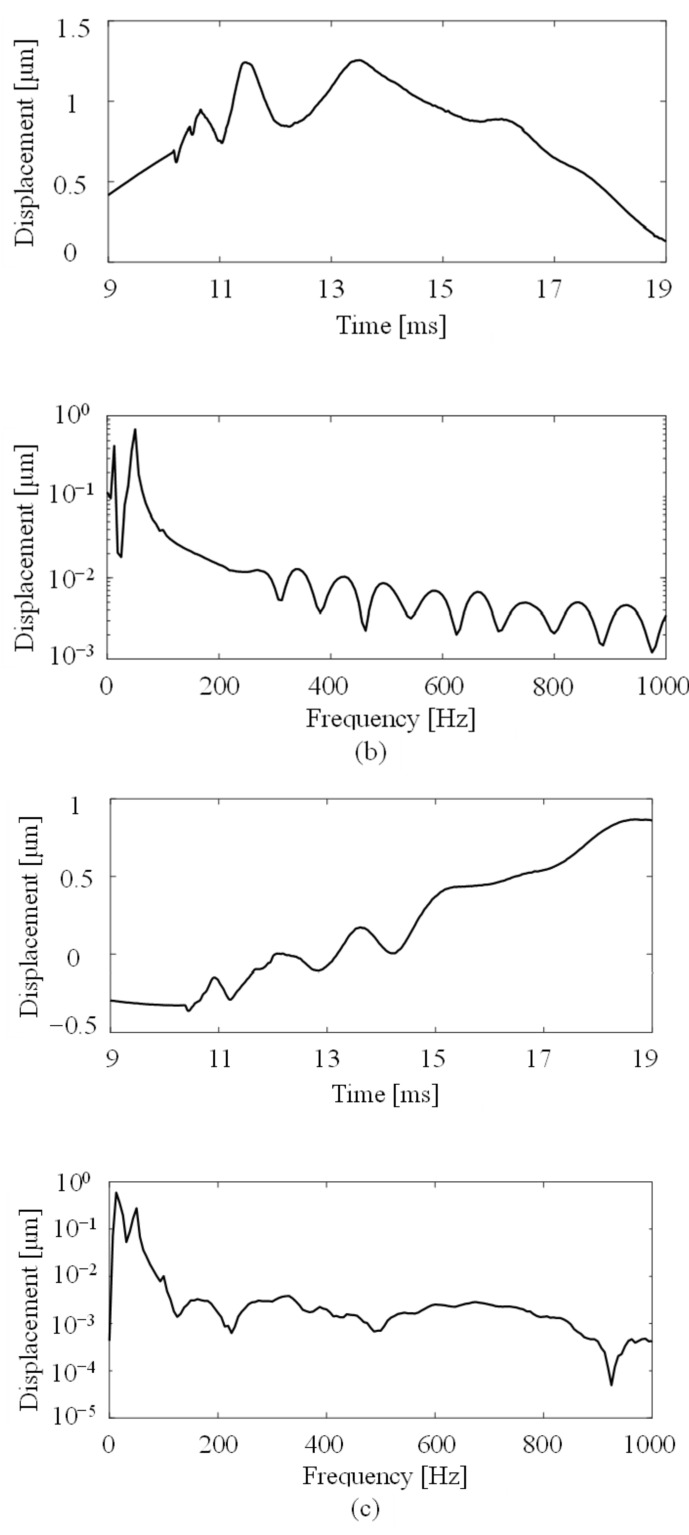
Time history responses and Fourier spectra of the measured displacement on mango 6 at (**a**) point 1, (**b**) point 12, and (**c**) point 24. (Measurement path: Line A).

Figure 3 shows the time history responses and the power spectra of the displacement on mango 6 measured by our system. We compared points 1, 12 (90° away from point 0), and 24 (180° from point 0). The Rayleigh wave was excited with an approximately 10 μm amplitude (Figure 3a). The amplitudes of the measured Rayleigh wave at points 12 and 24 were approximately one-tenth of the amplitude of the measured Rayleigh wave at point 1 (Figure 3b,c). In addition, the mangoes used in this experiment showed a strong viscoelastic feature because they were damped a few milliseconds after the excitation. The power spectra showed a 12.5 Hz peak frequency at points 12 and 24. This frequency was the so-called “rigid body mode”, where the mango vibrated without showing elastic deformation because it was seated on an air cushion. Additionally, the 50 Hz peak frequency appeared at points 1, 12, and 24 due to the power supply noise. For the above reasons, our system can generate a Rayleigh wave on a mango in a non-contact and non-destructive manner. On the other hand, the _0_S_2_ mode frequency, which has traditionally been used as a flesh firmness index for fruit, was not excited on the mangoes used in this experiment.

### 3.2. Detection of the Rayleigh Wave Propagation Velocity

**Figure 4:**
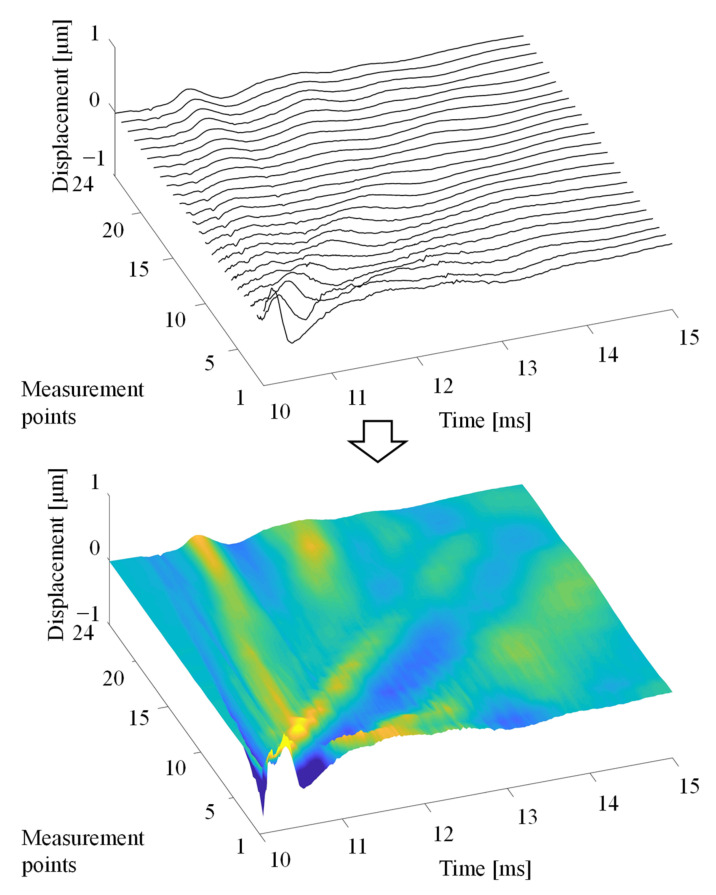
Visualization of Rayleigh wave propagation by arranging the time history responses of the measured displacement.

We calculated the Rayleigh wave propagation velocity generated by our system. To show how the Rayleigh wave propagates, Figure 4 plots the time history responses of the measured displacement at points 1–24 on the mango peel arranged in order of the measurement points. The horizontal line shows the time, while the vertical line shows measurement points, and the color describes the amplitude of the Rayleigh wave. The slope of a line is the Rayleigh wave propagation velocity, which was calculated by the least-squares method.

**Figure 5:**
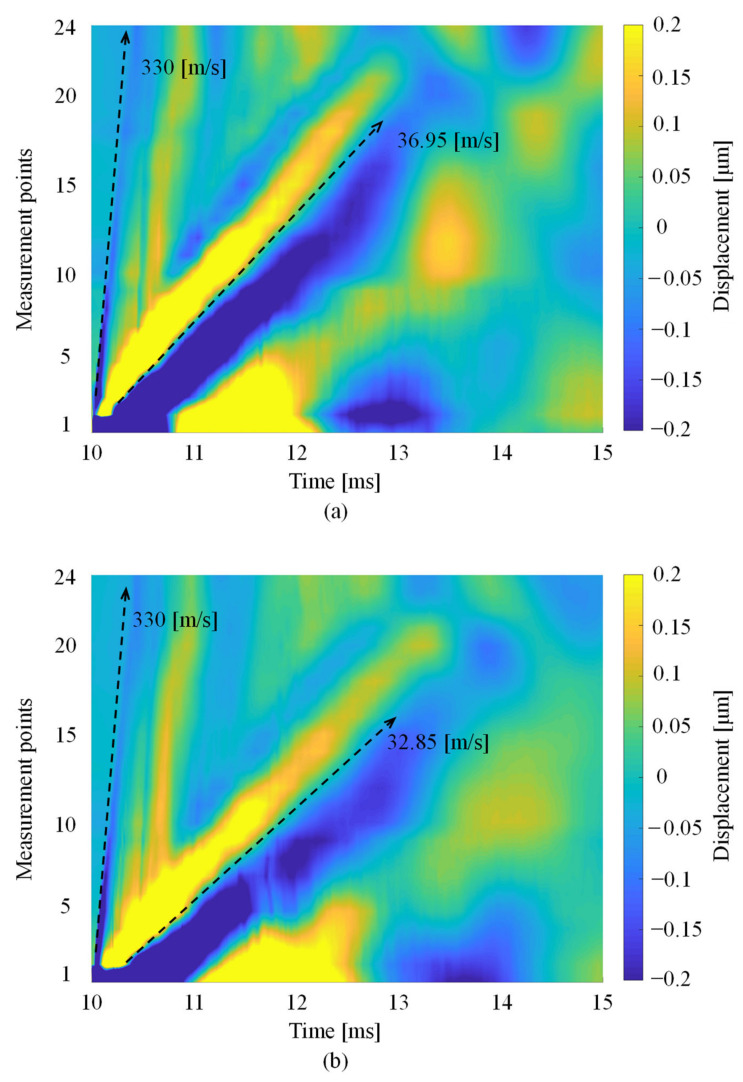
Rayleigh wave propagation on mango 6 on (**a**) Line A at 0 and (**b**) 216 h.

Figure 5 shows the Rayleigh wave propagation on mango 6 (Line A, 0 h). The line had two slopes, which were calculated as 330 and 37 m/s. Therefore, one LIP shock wave propagated on the mango peel, while the other was the Rayleigh wave generated on the mangoes.

### 3.3. Shift in the Rayleigh Wave Velocity of Mangoes during Storage

The Rayleigh wave propagation velocity *V* [m/s] is given by:

(2) $$V=\frac{0.87\sigma +1.12\sigma }{1+\sigma }\sqrt{\frac{G}{\rho }}$$

where *G* (Pa), *σ*, and *ρ* (kg/m^3^) are the shear elasticity, Poisson’s ratio, and density, respectively. The changes in *V* can be regarded as the changes in *G* if the changes in the density and Poisson’s ratio of mangoes are sufficiently small that they can be ignored during the storage period. According to [52], shear elasticity of fruit flesh is strongly correlated to firmness. Here, we consider the change in the firmness of mangoes as the change in the Rayleigh wave propagation velocity.

**Figure 6:**
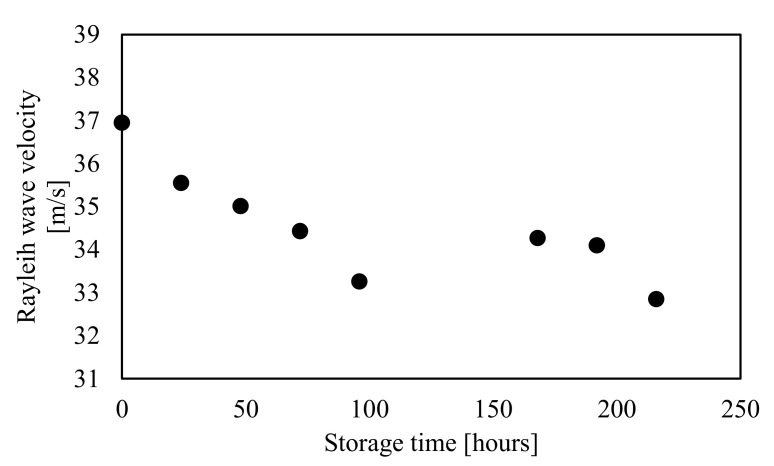
Relationship between the Rayleigh wave velocity and storage time of mango 6 (Measurement path: Line A).

Figure 6 shows the relationship between the Rayleigh wave propagation velocity and the storage time of mango 6 at Line A (Figure 2). The Rayleigh wave propagation velocity decreased during storage. The reduction rates of the Rayleigh wave propagation velocity after 96 and 216 h were approximately 10% and 1.2%, respectively. Therefore, we assumed that the changes in the firmness of mango 6 were nearly complete after 96 h of storage. Comparing the Rayleigh wave propagation velocities at 96 and 168 h revealed that the velocity increased by around 3% although the storage time increased. The increased velocity is attributed to the error related to installation of the LDV and the measurement points. Since we put reflection seals (approximately 3 mm × 3 mm) at each measurement point, the average circumference (270 mm) contained approximately ±6 mm error, indicating that the Rayleigh wave propagation velocity contained an approximately ±4% measurement error. In this experiment, mangoes with a change rate in the Rayleigh wave velocity within ±4% were not considered to have a change in the firmness as the threshold in the Rayleigh wave propagation velocity was ±4%.

**Figure 7:**
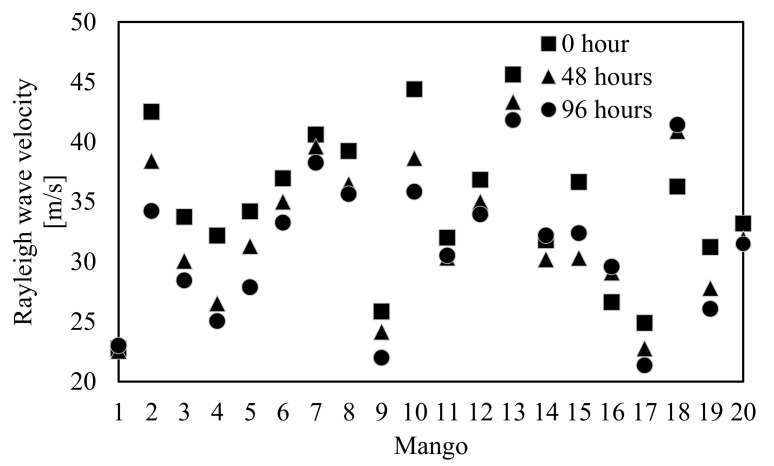
Shift in the Rayleigh wave velocity measured around Line A for mangoes 1–20 at 0, 48, and 96 h.

**Figure 8:**
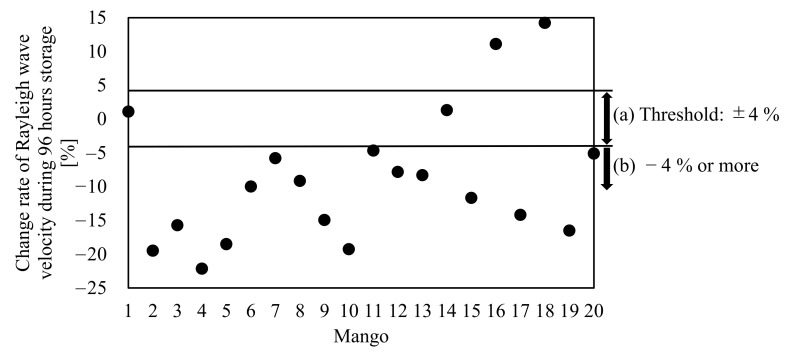
Change rate of the Rayleigh wave velocity measured around Line A of mangoes 1–20 after 96 h of storage. (**a**) Change rate of the Rayleigh wave is within the threshold. (**b**) Change rate of the Rayleigh wave exceeds the threshold.

**Figure 9:**
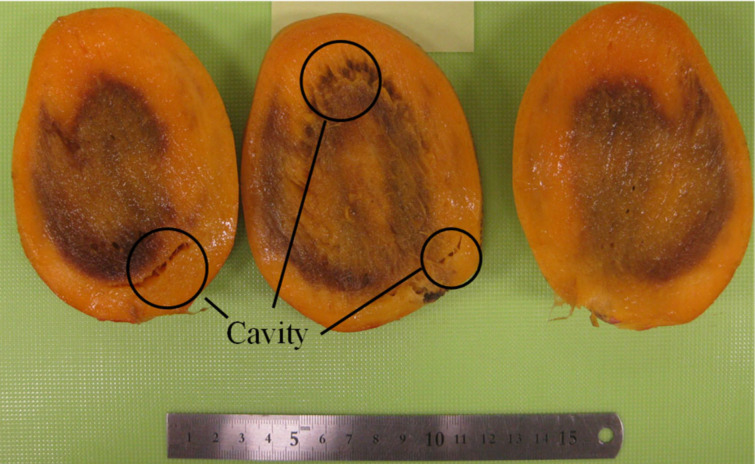
Cross-section picture that shows cavity decay (mango 16).

Figure 7 shows the Rayleigh wave propagation velocity on mangoes 1–20 measured around Line A at 0, 48, and 96 h. In addition, Figure 8 plots the change rate of the Rayleigh wave propagation velocity from 0 to 96 h, where the ±4% threshold is added as a reference. Almost all mangoes showed the same trend, where the Rayleigh wave propagation velocity decreased every 48 h (Figure 7). Although mangoes 1 and 14 slightly increased their Rayleigh wave propagation velocities, these were attributed to measurement error because the reduction rates of the Rayleigh wave propagation velocity for both mangoes were within the threshold (Figure 8). The Rayleigh wave propagation velocity increased by over 10% in 96 h for mangoes 16 and 18. Figure 9 shows cross-sectional pictures of mango 16, which were taken after the experiment. Mango 16 contained a cavity and showed some decay. Therefore, it is assumed that the Rayleigh wave propagation velocities of mangoes 16 and 18 increased due to the existence of the cavity near the measurement point or potential internal decay.

### 3.4. Effect of a Seed on Measuring the Rayleigh Wave Propagation Velocity

**Figure 10:**
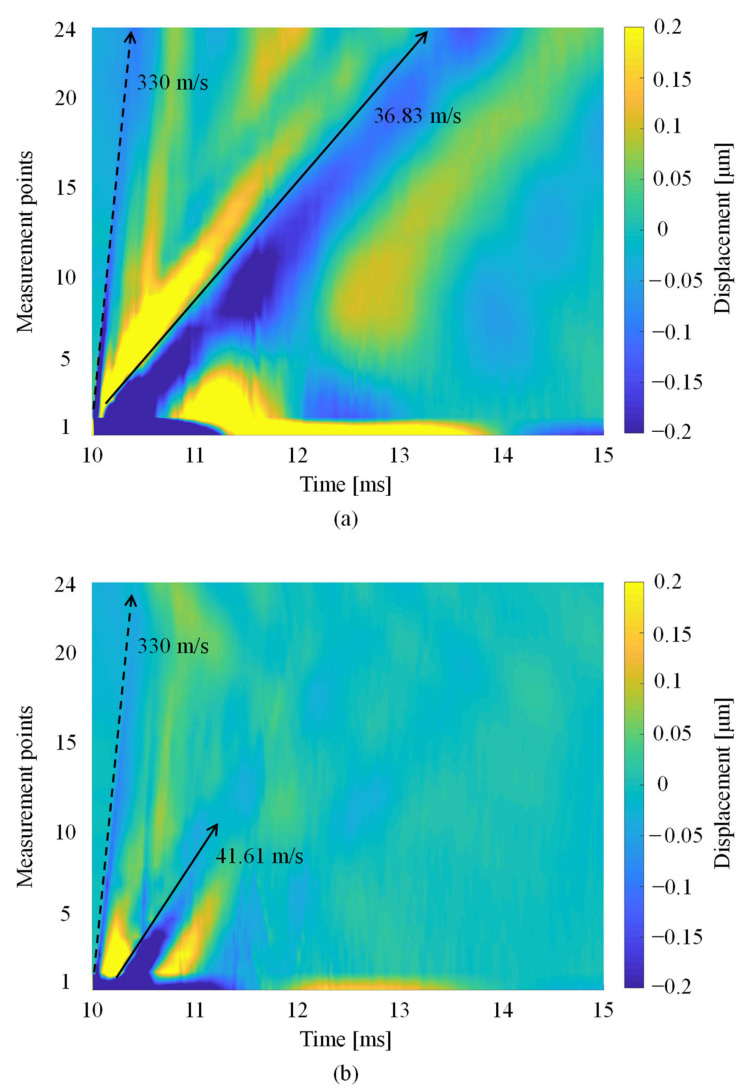
Rayleigh wave propagation on mango 12 at 0 h. (**a**) Line A where the measurement points are on the equator, (**b**) Line B where the measurement points are orthogonal to the equator.

We investigated the effect of a seed on the measurement of the Rayleigh wave propagation velocity to consider the reliability of our method. The Rayleigh wave velocities that propagated on Lines A and B (Figure 2b) of mangoes 9–20 were measured. Figure 10 shows the Rayleigh wave propagations on Lines A and B of mango 12 at 0 h. The Rayleigh wave propagated to point 24 on Line A, although it only propagated up to point 10 on Line B (Figure 10). Furthermore, the Rayleigh wave propagation velocities on Lines A and B were calculated as 36.8 and 41.6 m/s, respectively.

**Figure 11:**
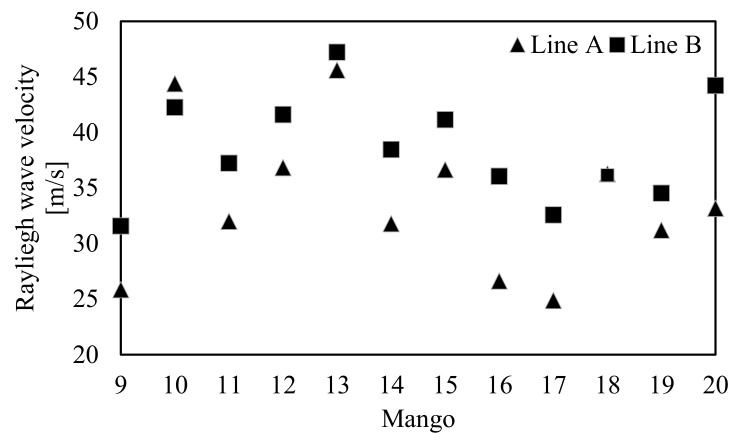
Rayleigh wave velocity measured around Line A and Line B of mangoes 9–20 at 0 h.

Figure 11 shows the Rayleigh wave propagation velocities on Lines A and B of mangoes 9–20 at 0 h. The propagation velocities on Line B were larger than those on Line A, except for mangoes 10 and 18. The propagation velocities of mangoes 10 and 18 on Line B were smaller than those on Line A, which can be explained by the measurement error because they are within the 4% threshold. A previous study found that the Rayleigh wave propagation velocity depended on the fruit flesh thickness [52]. Therefore, the mango seed might have affected the propagation velocity on Line B.

**Figure 12:**
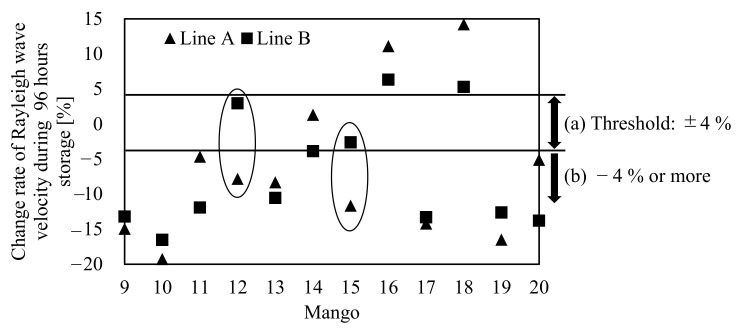
Change rate of Rayleigh wave velocity measured around Line A and Line B of mangoes 9–20 during 96-h storage. (**a**) Change rates of Rayleigh wave are within threshold. (**b**) Change rate of Rayleigh wave exceeds threshold.

Next, Figure 12 shows the change rate of the Rayleigh wave propagation velocities of mangoes 9–20 on Lines A and B. In Figure 12, the change rate of the propagation velocity was calculated using 0 and 96 h. The Rayleigh wave propagation velocities on Lines A and B showed the same trend, where the velocity decreased during storage. We could not detect a shift in the firmness of mangoes 12 and 15 on Line B because their change rates of the Rayleigh wave propagation velocity were within the 4% threshold. Nevertheless, the shifts in firmness of mangoes 12 and 15 were detected based on Line A. Mangoes 16 and 18 increased their propagation velocities on Lines A and B during storage, indicating that they might have had a cavity near the measurement point or potential decay inside. These results imply that the Rayleigh wave along Line A is less susceptible to seed-related effects and is better suited for the firmness assessment than Line B. In the future, we will investigate the locations of the measurement area for firmness assessment of mangoes in detail.

## 4. Conclusions

This work realized a firmness assessment for soft-flesh mangoes using a LIP shock wave excitation technique. LIP is generated near the excitation point on mangoes by condensing a high-output Nd:YAG pulsed laser.

The experiment, which employed 20 Kent mangoes as test materials, confirmed that our system can generate a Rayleigh wave on a mango fruit by applying an impulse excitation force based on the LIP shock wave excitation technique. Next, Rayleigh waves were generated on the mangoes during storage and their propagation velocity was investigated. The Rayleigh wave propagation velocity decreased as the storage time increased. The Rayleigh wave propagation velocity on the equator of Kent mangoes tended to decrease by over 4% in 96 h. The Rayleigh wave measured on two different lines propagated an independent distance and showed a different change rate of propagation velocity during 96-h storage.

The _0_S_2_ mode frequency, which is traditionally used for flesh fruit firmness assessments, was not excited on the mangoes used in the experiment. These results demonstrate that our system is effective for non-contact and non-destructive firmness assessments for fruit such as mangoes, which do not show the _0_S_2_ mode frequency.

We investigated the suitable measurement location for firmness assessment of mangoes. One was set on the equator, which is where the _0_S_2_ mode frequency is traditionally measured in fruit firmness assessments. The other was set on the orthogonal direction to the equator. If the firmness is assessed on the orthogonal direction to the equator, a mango may estimate more firmly than an actual state. This is because the Rayleigh wave propagation velocity on the orthogonal to the equator was larger than that on the equator. The reduction rate of the Rayleigh wave propagation velocity on the equator during storage tended to be greater than that on the orthogonal line to the equator. Hence, the Rayleigh wave propagation velocity should be measured on the equator for the firmness assessment of mangoes. In the future, we will consider in detail the point where a Rayleigh wave is excited (excitation point) and the measurement area where the wave propagates.

## Figures and Tables

**Table 1:** Mass, density, and circumference of mangoes.

Sample No.	Mass (g)	Density (kg/m^3^)	Circumference (m)
Average	457.0	1094	0.270
1	531.9	978.0	0.240
2	510.2	1001	0.300
3	493.9	1026	0.280
4	495.4	1064	0.298
5	493.1	990.0	0.300
6	496.7	1158	0.284
7	490.0	1174	0.284
8	501.8	741.0	0.277
9	437.6	1020	0.281
10	435.9	815.0	0.280
11	371.8	1039	0.253
12	371.7	1039	0.242
13	391.4	1094	0.262
14	401.2	1121	0.250
15	426.5	1192	0.240
16	438.9	1227	0.280
17	465.1	1300	0.300
18	398.3	1113	0.284
19	480.6	1343	0.281
20	519.6	1452	0.280

**Table 2:** Measurement paths and storage times of mangoes.

Sample No.	Measurement Path	Storage Time (h)
1–5, 7, 8	Line A	0, 48, 96
6	Line A	0, 24, 48, 72, 96, 168, 192, 216
9–20	Lines A and B	0, 48, 96
